# Breaking Electrochemical Scaling Laws in Atomically Engineered van der Waals Stack Multisite Edge Catalysts

**DOI:** 10.1021/acs.nanolett.5c03027

**Published:** 2025-07-28

**Authors:** Ding-Rui Chen, Jeyavelan Muthu, Jui-Teng Chang, Po-Han Lin, Yu-Xiang Chen, Farheen Khurshid, Hao-Ting Chin, Jing Kong, Mario Hofmann, Ya-Ping Hsieh

**Affiliations:** † 71556 Institute of Atomic and Molecular Sciences, Academia Sinica, Taipei 10617, Taiwan; ‡ Department of Electrical Engineering and Computer Science, 2167 2167 Massachusetts Institute of Technology (MIT), Cambridge, Massachusetts 02139, United States; § Department of Electronic Engineering, 34900 Chung Yuan Christian University, Taoyuan 320, Taiwan; ∥ Department of Low Dimensional Systems, J. Heyrovský Institute of Physical Chemistry, Prague 18223, Czech Republic; ⊥ Department of Physics, 33561 National Taiwan University, Taipei 10617, Taiwan; # Molecular Science and Technology Program, Taiwan International Graduate Program, Academia Sinica, Taipei 10617, Taiwan; ∇ International Graduate Program of Molecular Science and Technology, 33561 National Taiwan University, Taipei 10617, Taiwan

**Keywords:** Multisite catalysts, van der Waals stack edges, Hydrogen evolution reaction (HER), Overall water splitting, 2D materials

## Abstract

Electrocatalysis is key to sustainable energy conversion and storage, but its efficiency is limited by scaling laws between reactant adsorption and desorption. Multisite catalysts promises to overcome these limits, but challenges in fabrication and characterization hinder its validation. We present a platform to study and optimize multisite electrocatalysis. Leveraging van der Waals stacked 2D materials, we create catalytic edge assemblies with precise activity variations, enabling atomically engineered site separation and interaction. This approach enables the identification of multisite catalysts that enhance the hydrogen evolution reaction (HER) beyond single-site Sabatier scaling. Altering atomic-scale site separations reverts the system to single-site mechanisms, highlighting the importance of intermediate transport. Direct evidence of intermediate exchange is provided by electrostatic control of the sites, supported by ab initio simulations. We further engineer bifunctional catalysts for the oxygen evolution reaction (OER) and HER, achieving superior neutral water splitting. These findings enable the catalytic cascade design and complex electrochemical synthesis.

## Graphical abstract

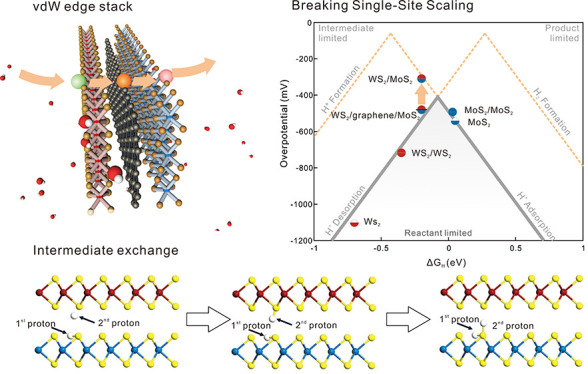

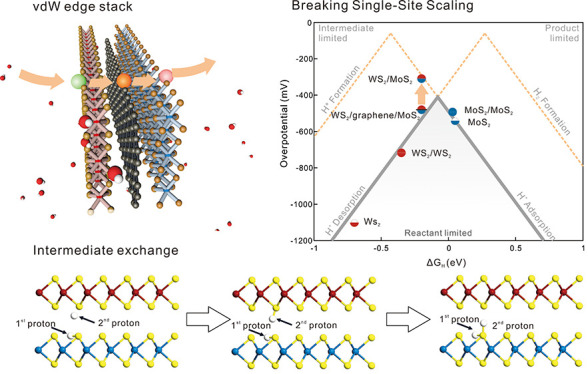

Electrocatalysis has recently received significant interest due to the promise of converting renewable energy into sustainable chemicals.^1^ Unfortunately, this vision is limited by the low efficiency of the electrochemical conversion process.^2^ Scaling relations between adsorption of reactants and desorption of products fundamentally restrain the energetics of electrocatalysis, and optimization has aimed at identifying the best trade-off between the two competing processes, leading to the well-known volcano plots.^3–5^

Theoretical work on gas-phase catalysis^6,7^ and organic catalysis^8,9^ have put forward a promising strategy to break these scaling laws. By separating adsorption and desorption to different catalytic sites, the energetics of both processes could be decoupled.^10^ Consequently, reaction yields could exceed the fundamental limit of single-site catalysis.^11^

Electrocatalysis has shown enhancements in multisite systems, such as catalyst alloys,^12,13^ catalyst/support systems,^14^ and materials heterojunctions.^15^ However, alternative mechanisms such as changes in morphology,^15^ conductivity^16^ hybridization,^17,18^ strain-effects,^19^ and electric fields^20^ could also explain the observations and definitive proof of multisite catalysis in electrochemistry remains elusive.^21–25^

Clear evidence of the synergetic interaction among different catalytic sites could be achieved by characterizing the transport of intermediate reaction products between them.^26,27^ Controlling the exchange of reactants, however, requires well-defined separation, exact composition, and rational hierarchy of different catalyst sites.^28^ Consequently, new strategies toward developing suitable model systems are required that combine large-scale crystallinity with atomically precise control over the placement and character of catalytic sites.^29^ Finally, each catalytic site should operate at its optimal electrostatic condition, necessitating complex electrical connectivity.^30,31^

We here introduce a powerful approach for realizing multisite electrochemical catalysis that achieves unprecedented precision and controllability. Our approach is leveraging the unique morphology of van der Waals (vdW) stack edges: whereas a crystal terminates in a homogeneous surface (Figure 1a), van der Waals stacks represent quasi-one-dimensional structures whose exposed surface consists of a compactly arranged edges of individual 2D materials layers. The variability of composition and atomic structure of different edges thus enables the realization of surfaces with abruptly varying catalytic activity that have no equivalent in nature (Figure 1b). Moreover, the proven ability to produce vdW stacks with arbitrary sequences of atomically thin 2D materials translates into the capacity to engineer the separation of catalytic sites with the ultimate precision.

**Figure 1:**
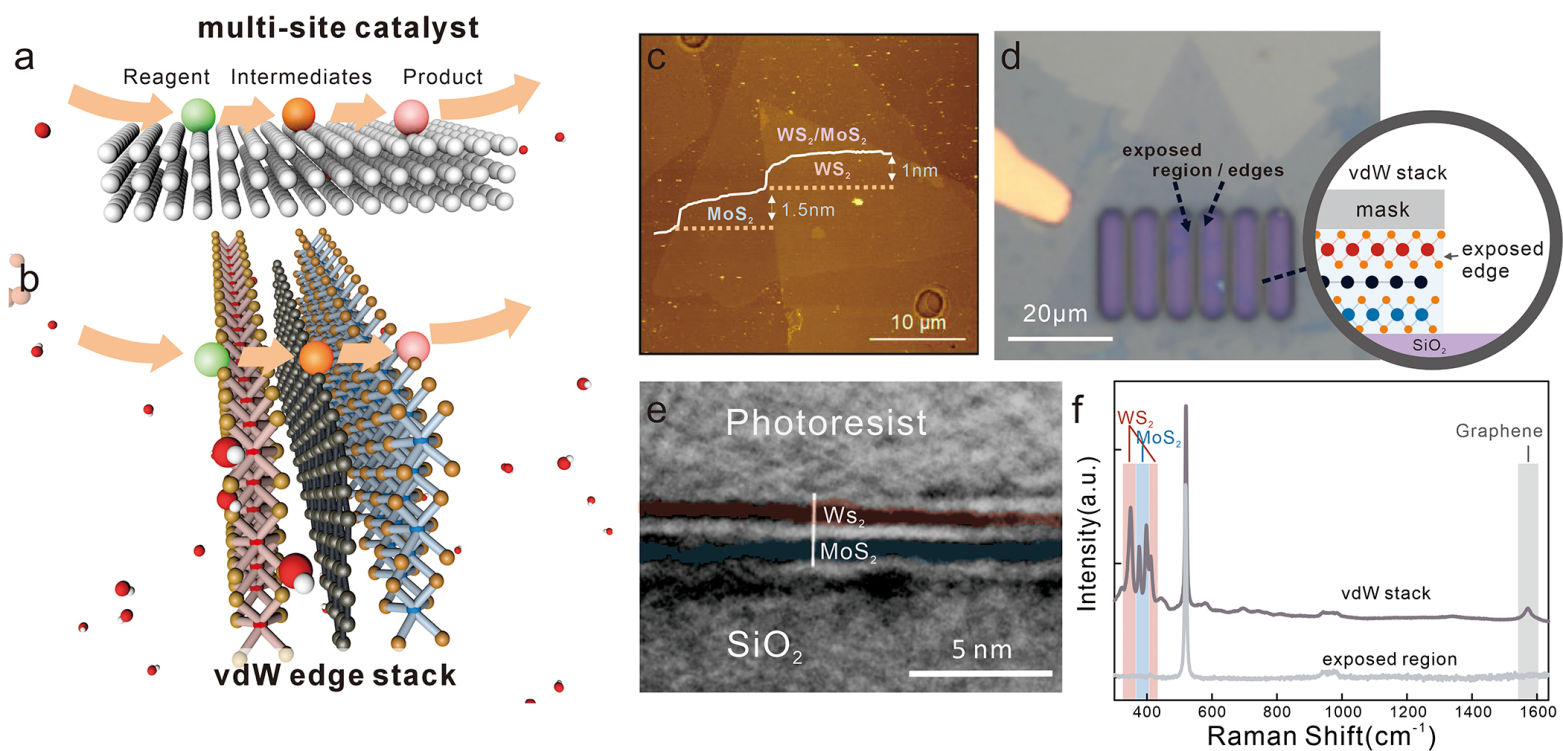
(a) Concept of multisite catalysis demonstrating the stepwise conversion of a reagent through exchange of intermediates between different catalytically active sites that are localized on the surface of a suitable support. (b) Proposed concept of multisite catalysis in vdW edge stacks showing the exchange of intermediate species at distinct 2D materials edges that are assembled in suitable sequences. (c) Atomic force micrograph (AFM) of CVD grown 2D materials that are stacked through wet-chemical transfer from their support to a SiO_2_ substrate. (d) Micrograph of photolithographically patterned windows to expose the edges of vdW stacks through plasma etching (the focal plane was chosen to be on the substrate, making the higher photoresist region look blurry). (inset) Schematic of exposed edges and photoresist-passivated basal plane of the vdW stack. (e) Cross-sectional HRTEM image of the resulting vdW stack edges in the case of a WS_2_/MoS_2_ stack. (f) Raman spectra demonstrating the response of the MoS_2_/graphene/WS_2_ vdW stack in (d) and the complete removal of the stack within the window of (d).

Using this novel capability, we identify specific combinations of active sites that yield hydrogen evolution reactions with a performance beyond single-site electrochemical scaling laws. Changing the separation between active sites by single-atomic distances reverts the reaction to a single-site catalytic mechanism, which demonstrates the sensitivity of the intermediate transport to multisite separation. Direct experimental evidence of intermediate exchange during hydrogen evolution reactions was achieved through electrostatic control of individual catalyst sites, and ab initio simulations confirm the observed control over the selectivity of the HER process. Atomic engineering of multisite catalysts for simultaneous OER and HER enables the realization of superior bifunctional multisite catalysts for neutral water splitting. Our results open exciting opportunities for the top-down design of catalytic cascades for future complex electrochemical synthesis processes.

2D material edges represent ideal electrocatalysts due to their simple crystalline composition, exact atomic bonding structure, and good conductivity. We bring edges into atomic proximity by combining multiple 2D material layers into a van der Waals stack. Each edge within the vdW stack exhibits reactive sites with specific affinities to reactants and intermediates, allowing us to engineer multisite functionalities by choosing appropriate stacking sequences (Figure 1b).

To realize such vdW stack edge catalysts, we first synthesize different 2D materials at large scale by chemical vapor deposition (CVD), as detailed in Methods in the Supporting Information. The grown materials are then transferred onto each other using an established wet transfer approach and characterized by optical spectroscopy (Figure S1).^32^ The success of this approach can be inferred from atomic force micrographs of partially overlapped layers, which show a height distribution, as expected for the stacking of single-atomic layers (Figure 1c). This assessment agrees with the Raman spectrum and selected area diffraction (SAED) analysis, which shows a twisting arrangement of individual monolayers (Figures S1 and S2). Raman and X-ray photoelectron spectroscopy (XPS) results confirm the preservation of the pristine structure with negligible oxidation after stacking, as shown in Figures S1 and S3.

We expose edges in these vdW stacks by photolithographic patterning (Figure 1d):^33^ A photoresist is exposed, developed, and patterned, followed by a 3 min oxygen plasma treatment to remove the basal plane of the 2D material within the exposed region. Previous reports suggested that this process can create TMD edges with specific terminations.^34^ Cross-sectional high-resolution transmission electron microscopy (TEM) shows the well-ordered stacking of continuous MoS_2_ and WS_2_ films with smooth and clean edges (Figure 1e), showing an interplanar spacing of 0.63 nm, consistent with typical TMD materials.^35–37^ Raman characterization further confirms the high quality of the material after patterning (Figure 1f). (For more detailed characterization, including Cross-Sectional TEM, Raman spectroscopy, Atomic Force Microscopy (AFM), and Photoluminescence (PL) spectroscopy, please refer to Figures S1–S6.)

The presented fabrication approach represents a universal method to realizing “vdW edge stacks” with particular suitability for electrochemical catalysis. Commonly, electrochemical measurements on 2D materials have to disentangle the contributions from edges and the portion of the 2D basal plane that is exposed to the electrolyte.^38^ In our case, the same photoresist window that is used to pattern the vdW stack edges also serves as a protective coating that prevents electrolyte interaction with the basal plane (inset of Figure 1d). The exposed window size was chosen to be 80 μm^2^ with a perimeter of 288 μm throughout all experiments to ensure that the total reaction current could be directly employed to compare different vdW stack edge combinations.

van der Waals stack edges with several different stacking sequences were investigated for their performance as electrochemical catalysts (Figure 2a). Hydrogen evolution reactions were chosen as an initial focus due to their importance for energy storage and the simplicity of the involved reaction steps. First, the edges of individual single layers were investigated. Compared to the exposed basal plane, single 2D material edges exhibit a significantly enhanced activity, as evidenced by an approximately 3-fold increase in the total reaction current of the HER for WS_2_ and a 260-fold increase for MoS_2_, despite the reduced reaction area (Figure S7), which agrees with previous reports on the enhanced electrochemical activity of 2D materials edges.^39^

**Figure 2:**
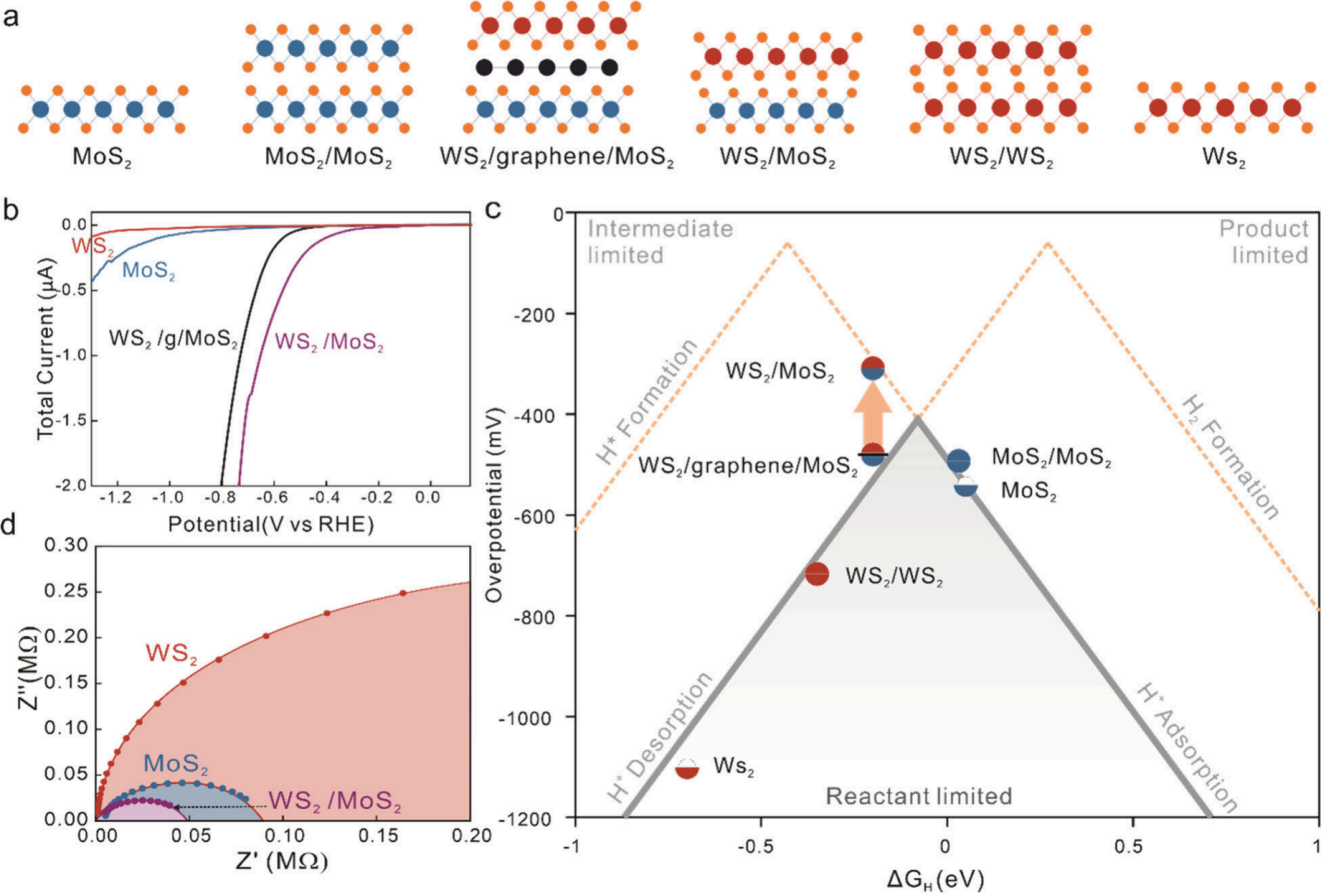
Electrochemical performance of multisite catalysts. (a) Schematic of investigated vdW stack edges covering homogeneous stacks and heterogeneous vdW stacks with two and three components. (b) Polarization curves of homogeneous and heterogeneous vdW edge stacks, (c) Extracted overpotential vs simulated adsorption energy for HER^23–25^ with the indication of scaling limitations arising from fundamental reaction steps of HER.^28^ The WS_2_/MoS_2_ stack shows a departure from the traditional volcano plot indicating the departure from proton adsorption and desorption. (d) Electrochemical impedance spectroscopy (EIS) characterization of homogeneous and heterogeneous vdW edge stacks.

Second, a larger HER reaction efficiency of MoS_2_ edges compared to WS_2_ edges was observed (Figure 2b) that can be explained by the Sabatier scaling laws.^40,41^ When the interaction between the catalyst and hydrogen is too weak, the low hydrogen concentration on the catalyst surface limits the reaction efficiency. When the interaction is too strong, the adsorbate remains on the catalyst and blocks the active site, thus providing a second limit on the reaction. This scaling relation between adsorption and desorption can be visualized by plotting the overpotential for both 2D materials edges against the Gibbs free energy of proton adsorption, and our results follow the commonly employed HER volcano plot (Figure 2c). MoS_2_ edges exhibit an interaction that represents a near-ideal trade-off between the two limits and consequently display a higher efficiency at hydrogen evolution than WS_2_.

With the performance of single layered TMDCs established, we produce homogeneous vdW stack edges by stacking MoS_2_ on MoS_2_ and WS_2_ on WS_2_. Both vdW stack edge systems exhibit a slightly enhanced HER (Figure S8) and follow theoretical predictions of an improved hydrogen adsorption on bilayer edges compared to their single-layer equivalent.^42,43^ The overpotentials of both vdW stack edge sequences follow the same volcano plot (Figure 2c), indicating the similarity of the underlying adsorption and desorption mechanism.

The main finding of our work is the surprising difference in the HER when certain vdW stack combinations of different 2D materials are employed. Compared to homogeneous edge stacks of bilayer MoS_2_ and bilayer WS_2_, the vdW edge stack consisting of a layer of WS_2_ and a layer of MoS_2_ shows a significant enhancement in the polarization curve in both overpotential and slope. (Detailed HER polarization curves for all stack configurations can be found in Figure S8.) When plotting the overpotential vs the calculated Gibbs free energy, we observe that its value deviates from the volcano-plot spanned by the homogeneous stacks (Figure 2c). This observation is the first hint that the scaling law which has controlled HER catalysts can be broken through multisite catalysts.

To confirm the occurrence of an intermediate exchange between active sites on MoS_2_ and WS_2_ edges, we modify the separation between these sites. For this purpose, graphene edge sites were introduced between the MoS_2_ and WS_2_ edges. The achieved composition and structure represent the most complex multisite HER catalyst to date.^44^ Despite the similarity in composition and superiority in carrier conduction, the ternary multisite catalyst system falls onto the conventional volcano plot (Figure 2c), indicating the return to the noninteracting catalytic site picture. These results provide additional experimental evidence that MoS_2_ and WS_2_ exhibit synergistic interactions that are suppressed upon extending their separation by a single lattice constant.^29,45^ The observed synergy agrees with theoretical predictions that optimization of adsorption sites for each intermediate reaction step could break single-site scaling laws.^46,47^

We further corroborate this initial evidence of multisite catalysis at WS_2_/MoS_2_ stack edges by impedance spectroscopy (Figure 2d). Compared to the homogeneous constituents, the WS_2_/MoS_2_ edge stack exhibits a 2-fold increase in the heterogeneous charge transfer reaction rate compared to MoS_2_ and a 10-fold improvement compared to WS_2_. These findings indicate that the presented multisite catalyst exhibits properties beyond the sum of its parts. The impact of multisite synergy is not limited to HER, and we have also demonstrate the enhancement of OER in WS_2_/MoS_2_ vdW heterostack edges compared to the individual constituents as detailed in Figure S9.

While the breaking of the scaling law provides initial experimental evidence of multisite catalysis, we proceed to find direct confirmation for the exchange of intermediates between catalytic sites. Toward this goal, we take advantage of another unique feature of vdW edge stacks: each layer within the vdW stack can be electrically contacted through its basal plane (inset of Figure 3a). The intra-plane conduction is more efficient than inter-plane hopping, permitting the independent electrostatic control of individual edge sites. (More details on the fabrication and electrical properties of electrically contacted vdW edges are provided in Figure S10.) This multicontact arrangement represents a significant advance over previous multisite electrocatalysts, where all sites share the same electrostatic potential.^24^

**Figure 3:**
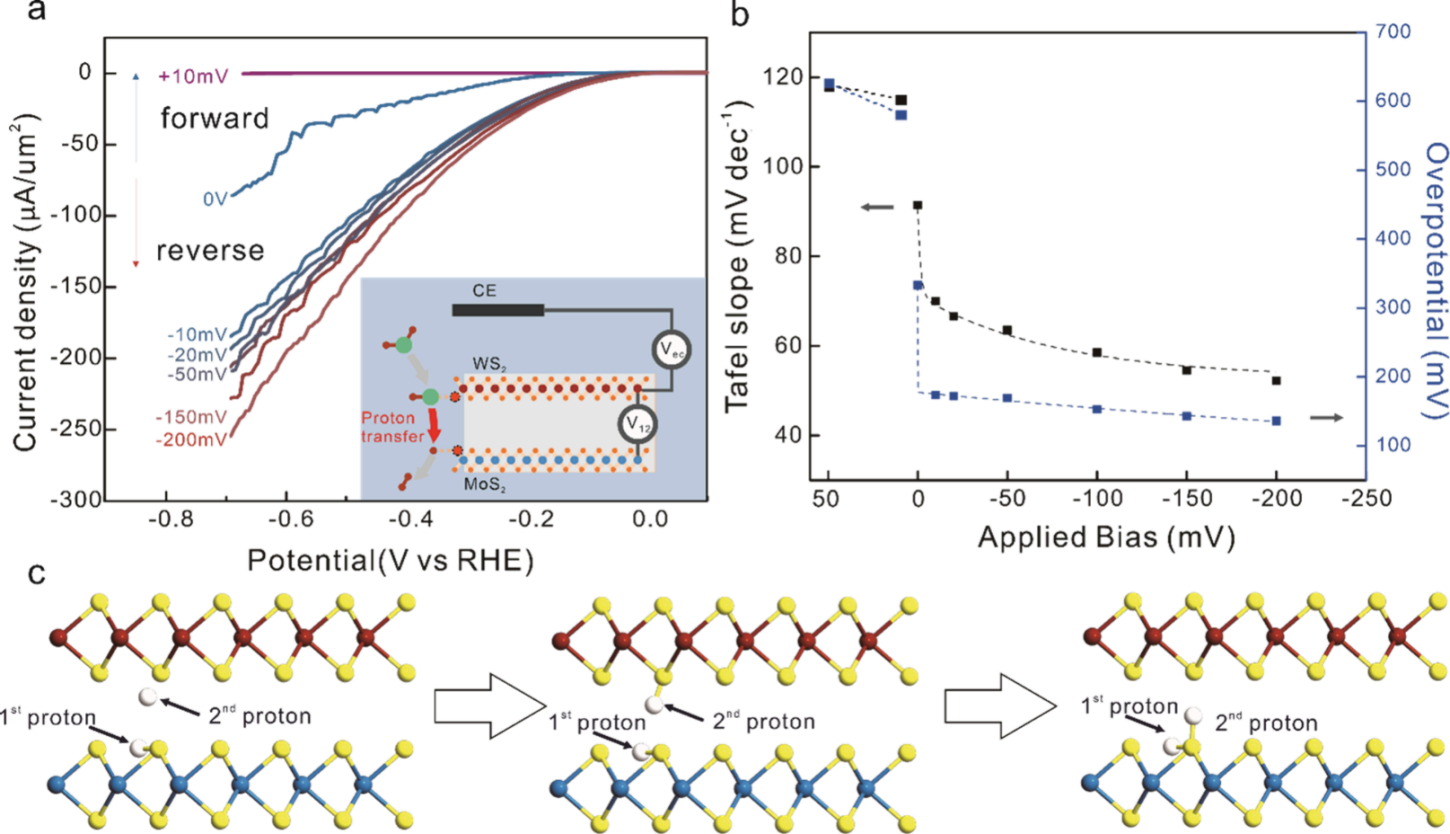
Investigation of multisite electrocatalysis through individually addressable sites. (a) HER polarization curves relative to the MOS_2_ potential at different potential differences between MoS_2_ and WS_2_. (inset) Concept of individual addressable catalytic sites with indication of applied potential differences and proposed proton transfer process. (b) Applied bias vs Tafel slope and overpotential at a reaction current density of −1000 mA/cm^2^. (c) Ab initio simulation results of structure-optimized hydrogen location on WS_2_/MoS_2_ for the HER demonstrating interaction of protons between adsorbed protons on MoS_2_ (bottom) and WS_2_ (top).

Using this arrangement, we investigate the intermediate transport between WS_2_ and MoS_2_ during HER. Hydrogen evolution was conducted, while a potential difference was applied between MoS_2_ and WS_2_ edges. At a potential difference of 0 V, the WS_2_ and MoS_2_ edges are at the same potential and the original polarization curve of the WS_2_/MoS_2_ stack is obtained. However, a significant change in the polarization curves is observed as the potential difference is increased (Figure 3a). If the WS_2_ is positively biased with respect to the MoS_2_, an enhanced reaction current is observed. A simple explanation that such effect originates from an averaging of the potentials between both components^48^ is disproved by our observation that the HER overpotential changes by 15 mV per mV of potential difference (Figure 3b). Instead, the trends confirm a concerted exchange of intermediates between WS_2_ and MoS_2_ during the HER process: the strong adsorption energy of protons on WS_2_ causes a preferential attachment to this site. Upon positive biasing, the adsorbed protons experience a force from WS_2_ to MoS_2_. The HER is completed by the desorption from MoS_2_ (inset of Figure 3a). The field-induced drift of adsorbed protons between WS_2_ and MoS_2_ confirms the shuttling of intermediates, which is clear evidence of multisite functionality. The proposed shuttling of intermediates is further confirmed by reversing the potential difference between the edge sites. If a negative bias is applied to the WS_2_, adsorbed protons are confined to the WS_2_ edge and a significant decrease in reaction current is observed.

We conduct ab initio simulations to investigate the intermediate transfer between vdW edge stack multisites during the HER process. For this purpose, we extend conventional simulation approaches that only consider the adsorption and desorption of a single proton at one active site.^49^ Instead, we introduce interaction with a second adsorbed proton to account for the experimentally observed Tafel reaction regime.^50^ We initially relax the first proton and observe that the lowest energy position is obtained by bonding it with a sulfur atom in the MoS_2_ edge. This structure resembles the proton adsorption in the bare MoS_2_ case from previous calculations.^50^ A second proton is then introduced, which is found to adsorb on the WS_2_ sulfur site. Subsequently, its bond reorients to bring the second proton in close proximity to the first proton and initiate bonding between them, allowing the formed molecule to desorb (Figure 3c). This sequence of steps agrees with our experimental observations.

The emergence of a fundamentally different proton adsorption mechanism for the heterostructure demonstrates the importance of the proton exchange between the distinct catalytic sites. Experimental and theoretical characterization confirm that the decoupling of adsorption and desorption steps leads to higher activity than homogeneous bilayers such as 2L MoS_2_ or WS_2_, as evidenced by experimental data in Figure S8.

Electrostatic adjustment of the intermediate exchange pathways is further shown to provide control over the reaction selectivity. Despite its simplicity, the HER exhibits two possible reaction pathways. The Tafel pathway signifies the formation of molecular hydrogen through the reaction of two surface-adsorbed protons, whereas the Heyrovsky pathway utilizes one adsorbed proton and a solvated proton. The selectivity of HER toward one of the processes can be inferred from the Tafel slope.^51^ Upon application of an electrostatic difference between the MoS_2_ and WS_2_ layer, we observe a monotonic decrease of the Tafel slope from ∼100 mV/dec to ∼40 mV/dec (Figure 3b). This behavior suggests that a small electrostatic modification can transition the HER process from the Volmer regime to the Heyrovsky regime.^51^ In addition to demonstrating the fundamental change in electrochemical reaction mechanisms in multisite catalysts, the observed Tafel slope represents the lowest value for any reported 2D material (Table T1 in the Supporting Information).

Our results not only validate the multisite catalytic ability of vdW stack edges but also demonstrate the potential of electrically modifying the electrostatic environment of individual catalyst sites in atomic proximity as a novel degree of freedom toward controlling and optimizing complex catalytic processes. Furthermore, the observed abrupt change in current upon biasing opens up new routes to switching electrochemical transistor devices for future computing.^52^

Finally, we demonstrate the potential of atomically engineered multisite catalysts for more complex electrochemical reactions. The presented control over reaction pathways provides opportunities to judiciously design catalytic processes with unprecedented control over selectivity and efficiency. We illustrate this ability by conducting neutral pH water splitting due to the importance of research on this topic for sustainable energy production and storage.^53,54^ Compared to HER, this process exhibits multiple intermediates and parallel reaction processes.^55–57^

We investigated all permutations of homogeneous and heterogeneous vdW stack edges for their utility in the cathodic and anodic half-cell reactions (Figure 4a). Surprisingly, we observe that WS_2_/MoS_2_ heterostack edges would outperform all other combinations as both the anode and cathode in both the OER and HER form an efficient bifunctional catalyst for overall water splitting.

**Figure 4:**
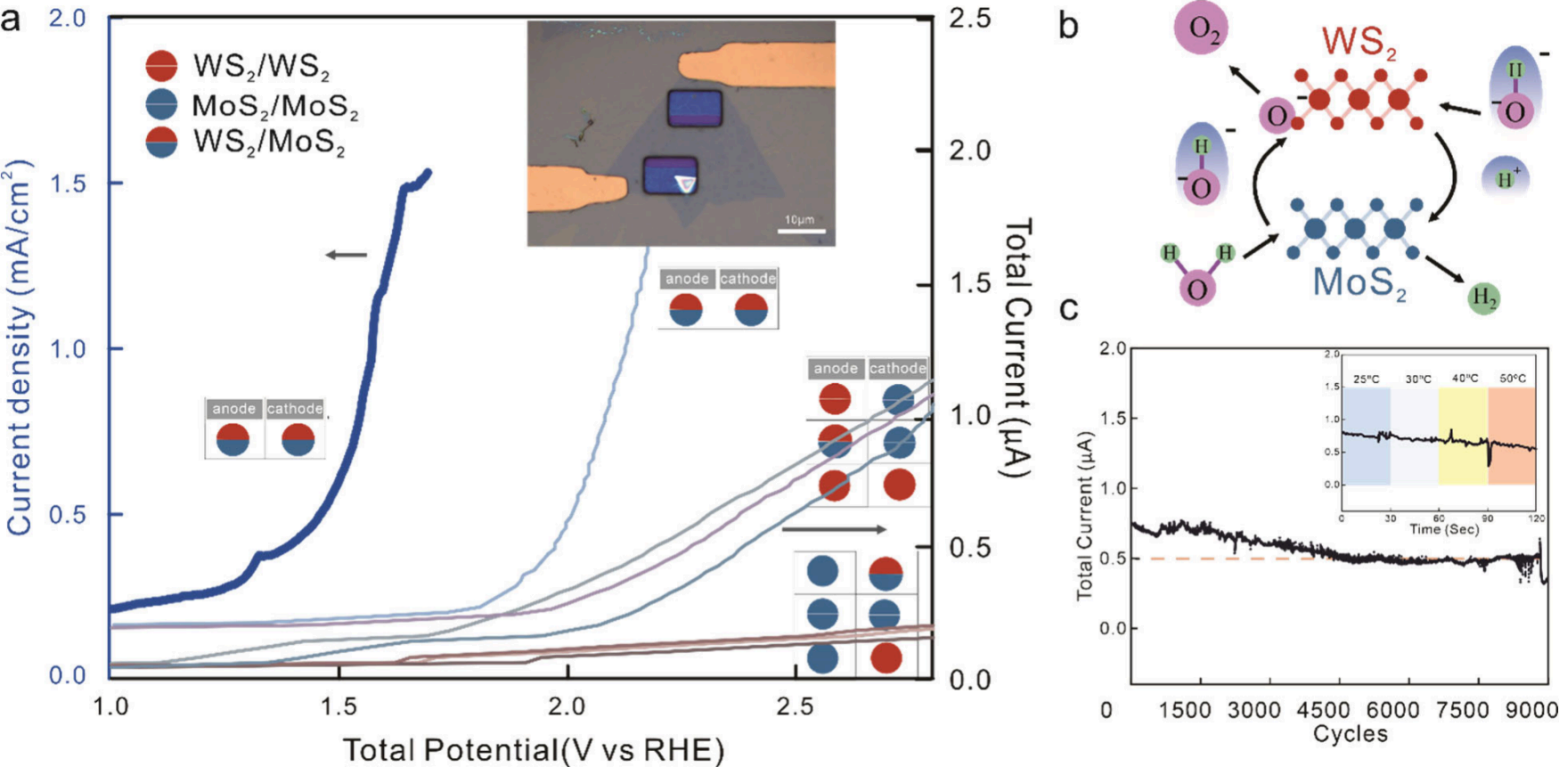
(a) Overall water splitting abilities for different vdW stack edges utilized as anode or cathode, evaluated by combining OER and HER half-cell reactions (axis on right) and by conducting total water splitting on WS_2_/MoS_2_ system (axis on left). (inset) Optical micrograph of the overall water-splitting WS_2_/MoS_2_ microreactor. (b) Proposed multisite mechanism resulting in overall water splitting. (c) Long-term stability tests conducted over 5000 cycles (inset), with evolution of reaction current at high overpotential and under various temperatures demonstrating high robustness. The initial fluctuation in current density is considered normal, attributed to the temporary blocking of the cathode side by the formation of hydrogen bubbles.^66,67^

To confirm this prediction, we fabricated a microreactor for neutral water splitting from WS_2_/MoS_2_ vdW stack edges. The utilization of a common electrode composition for both cathode and anode enables the aggressive scaling of the microreactor toward 15 × 55 μm overall size (inset Figure 4a)—which is one of the smallest electrochemical reactors to date.^38^ The resulting polarization curve represents the combined total reaction current for the OER and HER and demonstrates a clear overpotential of 1.51 V at 1 mA cm^–2^, which represents a superior performance compared to previous bifunctional catalysts (see Figure S11 for a comparison to literature).

Based on previously calculated adsorption energies,^58^ the multisite catalytic bifunctionality toward OER and HER can be understood as follows: Water will preferentially adsorb on MoS_2_ and OH^–^ is free to move to WS_2_. Oxygen will be bonded more strongly onto WS_2_ and serve as an anchor for the conversion to molecular oxygen. Conversely, the HER proceeds through the exchange of adsorbed protons from WS_2_ to MoS_2_ as previously described (Figure 4b). The complementary preference of OER and HER to the vdW edge sites impart our multisite catalyst with superior neutral water splitting performance that shows a 20% increase in power efficiency over commercial electrolyzers.^59^

Multisite catalysts not only exhibit a higher efficiency but the distribution of catalytic processes at different locations enhances the selectivity and robustness to catalyst deactivation.^60^ Indeed, we observe that the microreactor electrolyzer exhibits excellent stability, maintaining a steady current density over 5000 cycles at an above-driven voltage of 1.6 V (Figure 4c), exceeding cycle numbers reported in other electrocatalyst studies under similar pH conditions, indicating no notable oxidation despite prolonged operation.^61–64^ Additionally, the stability at increased temperatures was demonstrated (inset of Figure 4c), which corroborates the suitability for water splitting under realistic conditions.^65^ These findings highlight the promising potential of WS_2_/MoS_2_ vdW stack edges as a cost-effective substitute for precious metals in water electrocatalytic systems and pave the way for further advancements in clean and sustainable energy conversion technologies.

In conclusion, we have demonstrated a novel approach to engineer multisite catalysts that can break conventional scaling laws that limit the efficiency of important electrochemical reactions. By combining and patterning 2D materials into vdW edge stacks, specific catalytic sites can be assembled with atomic precision, and their electrochemical response can be individually controlled. These unique catalytic structures provide a powerful method to investigate and optimize the impact of multisite synergy on the yield and selectivity of catalytic processes. Our results opens up a route to tailoring the energetics and kinetics of catalysts to achieve complex electrochemical reactions with unequaled selectivity and efficiency.

## Supplementary Material



## Notes

Methods, additional figures, and a table (PDF)
