# Supplementary material for: Breaking Electrochemical Scaling Laws in Atomically Engineered van der Waals Stack Multisite Edge Catalysts
Source: Nano Lett. 2025 Jul 28;25(31):12059–66. doi: 10.1021/acs.nanolett.5c03027 (PMC12333411; doi:10.1021/acs.nanolett.5c03027)
Supplement: Supplementary file 1 [file nl5c03027_si_001.pdf]

Support Information For

Breaking electrochemical scaling laws in atomically engineered van-der-Waals stack multisite edge catalysts

Ding-Rui Chen [a,b,c,*], Jeyavelan Muthu [d], Jui-Teng Chang [a], Po-Han Lin [a,e], Yu-Xiang Chen [a,f,g], Farheen Khurshid [d], Hao-Ting Chin [a], Jing Kong [b], Mario Hofmann [d], and Ya-Ping Hsieh [a,f,g*]

[a] Institute of Atomic and Molecular Sciences, Academia Sinica, Taipei, 10617, Taiwan

[b]Department of Electrical Engineering and Computer Science, Massachusetts Institute of Technology (MIT), Cambridge, Massachusetts, 02139, USA

[c]Department of Electronic Engineering, Chung Yuan Christian University, Taoyuan 320, Taiwan

[d]Department of Low Dimensional Systems, J. Heyrovský Institute of Physical Chemistry, Prague, 18223, Czech Republic

[e]Department of Physics, National Taiwan University, Taipei, 10617, Taiwan

[f]Molecular Science and Technology Program, Taiwan International Graduate Program, Academia Sinica, Taipei, 10617, Taiwan

[g]International Graduate Program of Molecular Science and Technology, National Taiwan University, Taipei, 10617, Taiwan

The Supporting Information includes:

Supporting Information Text:
Methods
Figure S1 to S12
Table S1 to S1
References (1–49)

Methods

Material preparation

Monolayer MoS_2 and WS_2 were synthesized using the CVD approach, as described in our previous publications.[1, 2] Initially, molybdenum trioxide (MoO_3) and tungsten trioxide (MoO_3) thin films were deposited on graphite foil using an e-beam evaporator, acting as the molybdenum and tungsten source, respectively. A substrate was pretreated with a sodium chloride solution (consisting of 0.01 g ml^{-1} NaCl and 2.5×10^{-4} M NaOH) to enhance coverage during growth. These films were placed facing down on a Si/SiO_2 wafer at the center of a 1" tube within a clamshell furnace.

MoS_2 was grown in a two-step heating process: first reaching 700 °C in 20 minutes under Ar atmosphere, then increasing to 900 °C in 20 minutes while introducing 300 sccm H_2S (1% H_2S + 99% Ar) for 10 minutes. Finally, samples were cooled to room temperature.

After MoS_2 growth, we assessed the sulfur (S) vacancy density in our samples. According to our previous study,[3] CVD-grown TMDs typically exhibit 13% S vacancies, a value closely matching the 15% S vacancy density (1:1.688 ratio) observed in our samples after etching. This consistency confirms that the regions covered by the photoresist mask remained intact and were not damaged during the etching process.

Similarly, WS_2 growth followed a two-step heating process. Initially, the temperature was raised to 700 °C for 15 minutes under Ar flow. Next, the temperature was increased from 700 to 915 degrees in 20 minutes. During this stage, 100 sccm of H_2S (1% H_2S + 99% Ar) was introduced for 10 minutes at 760 mTorr. After the growth process, the samples were cooled down naturally.

Characterizations

Atomic force microscopy (AFM) results were obtained using a Bruker Dimension Icon and analyzed by Gwyddion sofware. Raman and PL measurements were performed in a home-built micro-Raman system with 532 nm excitation. Microscopic analysis, selected area electron diffraction (SAED) and Energy-Dispersive X-ray Spectroscopy (EDS) mapping of the heterojunction were conducted in a high-resolution transmission electron microscopy system (JEM2100F). Cross-sectional HR-TEM specimens were prepared by FIB etching (Hitachi NX2000) on copper grid. CrystalMaker software package was utilized to simulate the intensity ratio based on their configurations in order to unambiguously assign the identities of the SAED patterns.

Electrochemical measurement

Electrochemical measurements were performed using a microdroplet-based three-electrode system in a CH electrochemical workstation that was reported previously[4]. The HER was studied in an 0.5 M H_2SO_4 (acidic electrolyte), while the OER and full-

cell water spitting were investigated in a neutral medium with 1 M PBS (Phosphate Buffer Solution). The counter electrode was a Pt wire, while an Ag/AgCl electrode served as the reference electrode. The working electrode was created using a 70nm gold pad on the sample produced through standard photolithography and lift-off procedures. To ensure that the measured activity originated only from the exposed area, the gold contact and other non-exposed area were fully covered with photoresist by lithography. To validate HER measurements and sample preparation, we tested a bare SiO_2/Si substrate under the same conditions. The current density from the substrate was four orders smaller (~pA level) than that collected from 2D material, ensuring accurate results for HER. Electrochemical impedance spectroscopy was conducted at frequencies ranging from 1 Hz to 1MHz, commencing with an initial voltage of -0.5 V in a 0.5 M H_2SO_4 solution. Linear sweep voltammetry (LSV) was performed at a scan rate of 1 mV s^{-1}. To accurately evaluate the HER kinetics, all LSV curves were iR-corrected following standard procedures.[5] The electrochemical measurements were conducted multiple times to confirm consistent trends, following standard practices in micro-electrochemical studies.

The potential values reported in our manuscripts were referenced to the reversible hydrogen electrode (RHE) using the following equation:

$$E(RHE) = E(Ag/AgCl) + 0.197\ V + Vquasi + 0.05\ pH \quad (1)$$

The B2912A Precision Source/Measure Unit was utilized for measuring electrical transport characteristics and acting as a voltage supply to apply bias voltage to WS_2, in conjunction with an electrochemical workstation.

Density Functional Theory (DFT) Calculations

To understand the enhancement of heterogeneous vdW edges in HER compared to single-site catalysts, we conduct ab-initio simulations of the hydrogen evolution reaction process using density functional theory (DFT) on WS_2/MoS_2 structure, which is implemented in the QuantumATK Simulation Package. The convergence criteria for the calculations were set to 0.05 eV $Å^{-1}$ for the forces, with a plane-wave energy cut-off of 500 eV and a Gaussian smearing of 0.05 eV. The Brillouin zone was sampled using a 4×3×1 k-point grid for the supercell.

In our model, we employed zigzag edges terminated with sulfur atoms, which have been widely reported as the most catalytically active and energetically favorable configurations for MoS_2 and WS_2 edges,[6, 7] compared to armchair terminations.

To understand the different reaction pathways, we also calculated the free energy change associated with the first Volmer step , as shown in Figure S12. The hydrogen adsorption free energy (ΔG_H*) was determined using the following equation:

$$\Delta G_H^* = \Delta E_H^* + \Delta E_{ZPE} - T\Delta S \quad (2)$$

Here, Δ_{EZPE}−TΔS is about 0.24 eV. The hydrogen adsorption energyΔE_H* is calculated as

$\Delta E_H^* = E_{substrate+H} - E_{substrate} - E_{H2/2}$. (3)

In this equation, $E_{substrate+H}$ and $E_{substrate}$ denote the total energies of the substrate unit cell with and without an adsorbed hydrogen atom, respectively, while $E_{H2/2}$ represents the energy of one atomic hydrogen molecule.

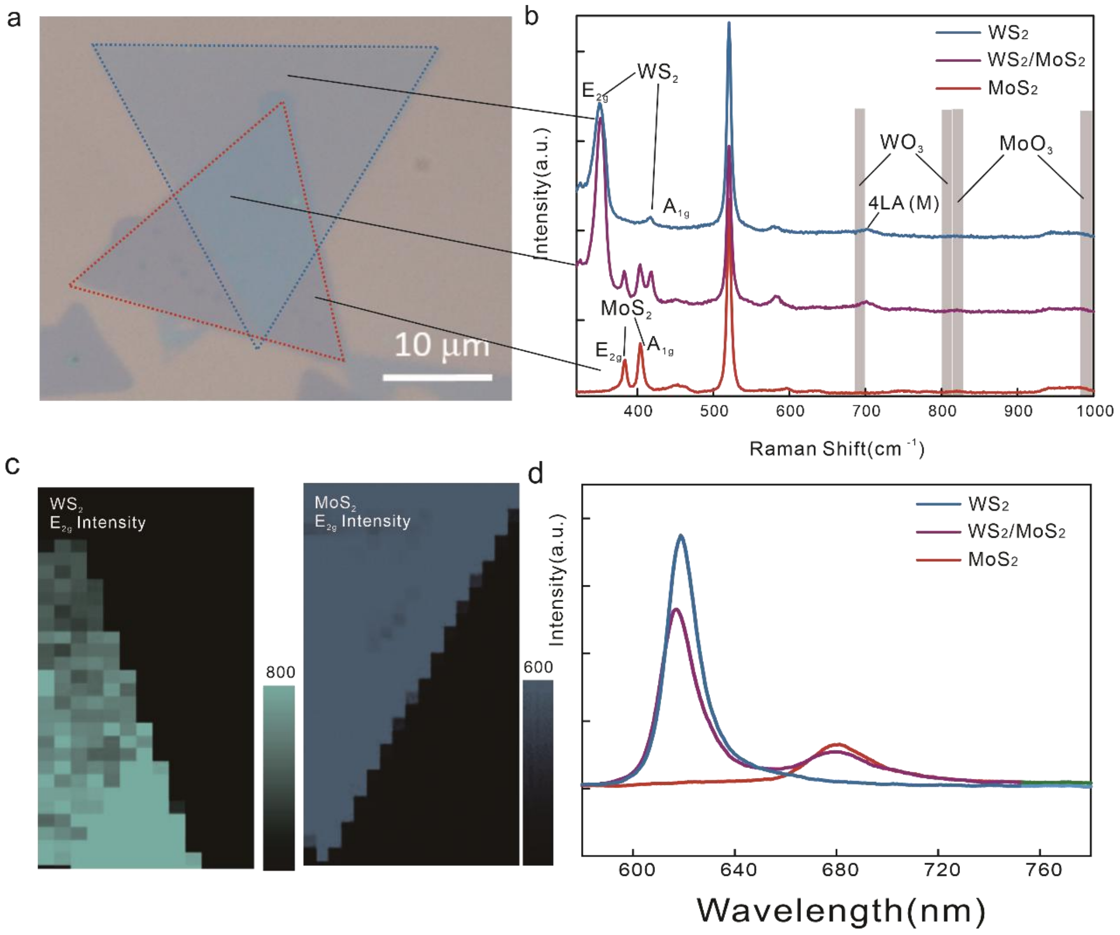

Supporting Information Figure. S1.| Fabrication of 2D vertical Heterostructures via Wet Transfer

(a) Optical microscope image showing triangular WS_2 and MoS_2 regions, marked by blue and green dashed lines, forming the vertical heterostructure.

(b) Raman spectra confirm the structural integrity and oxidation states of the WS_2/MoS_2 stack. Raman peaks of WS_2 (E_{2g} at 351.4 cm^{-1}, A_{1g} at 417.3 cm^{-1})[8] and MoS_2 (E_{2g} at 383.8 cm^{-1}, A_{1g} at 417.3 cm^{-1}).[9] The narrow vibrational frequency difference of WS_2 (Δ~ 65.9 cm^{-1}) and MoS_2 (Δ~20 cm^{-1}) between E_{2g} and A_{1g} (~404 cm^{-1}) confirmed the monolayer sheet structure.[8, 10] Notably, no apparent Raman signals associated with oxidized byproducts such as WO_3 (698, 809 cm^{-1}) or MoO_3 (820, 995 cm^{-1}) were detected, indicating minimal oxidation during the transfer process.[11, 12] A weak peak at 701 cm^{-1} is attributed to the 4LA(M) mode.[13]

(c) Raman E_{2g} intensity mapping confirms well-stitched interface between the two layers and crystallinity.

(d) PL spectra show emission at 618 nm (WS_2) and 680 nm (MoS_2), with the WS_2/MoS_2 heterostructure displaying slightly weaker peaks due to energy and charge transfer in the type II heterojunction.[14]

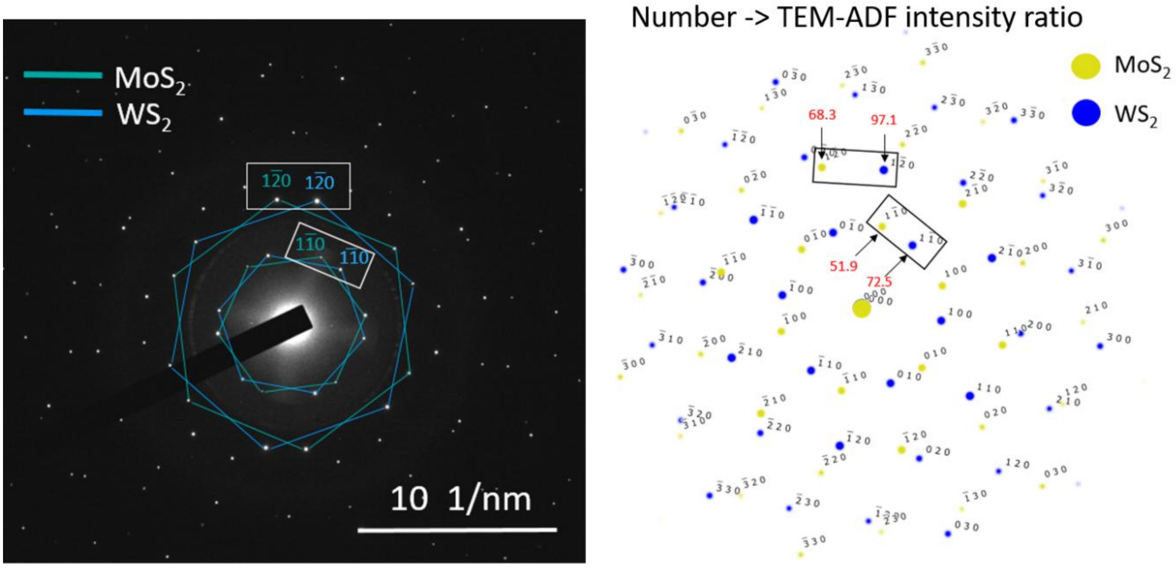

Supporting Information Figure. S2.| Identifying WS_2/MoS_2 SAED patterns: Experimental and Simulation Analyses. The selected area diffraction (SAED) patterns from a copper grid revealed four sets of 6-fold symmetric diffraction patterns. Two sets showed a lattice spacing of 0.27 nm, and two others had 0.158 nm, consistent with WS_2 and MoS_2, confirming a well-crystallized heterostructure.[15, 16] Simulation via Crystalmaker software confirmed that WS_2 has higher ADF intensity at the [$1\bar{1}0$]and [$1\bar{2}0$] zone axes compared to MoS_2. Combining experimental and simulation analyses enabled precise identification of the honeycomb SAED structures of both materials.

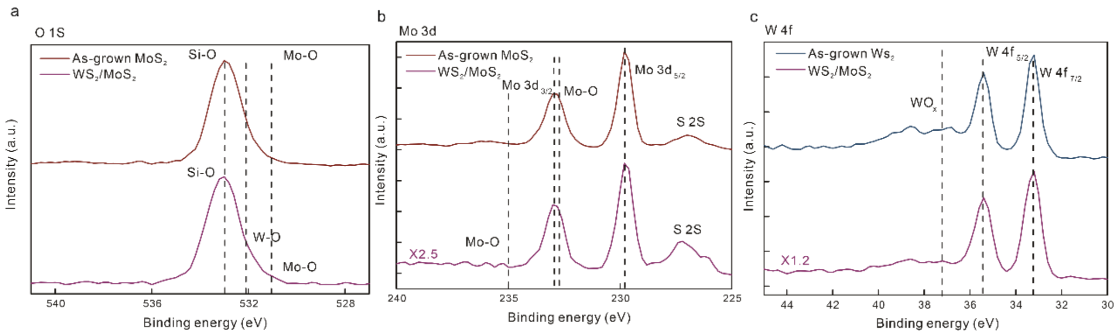

Supporting Information Figure. S3.| X-ray photoelectron spectroscopy (XPS) of as-grown and stacked samples: (a) O 1s (MoS_2, WS_2/MoS_2), (b) Mo 3d (MoS_2, WS_2/MoS_2), and (c) W 4f (WS_2, WS_2/MoS_2). XPS analysis was performed to evaluate the chemical composition and oxidation states of the stacked WS_2/MoS_2 heterostructure. In the Mo 3d region, two distinct peaks at 233.5 eV ($3d_{5/2}$) and 236.5 eV ($3d_{3/2}$) confirm the presence of MoS_2,[17, 18] while the W 4f region shows characteristic WS_2 peaks at 33 eV ($4f_{7/2}$) and 35 eV ($4f_{5/2}$).[19, 20] The O 1s, Mo 3d, and W 4f spectra of the WS_2/MoS_2 samples show no detectable Mo–O or W–O peaks, and the binding energies remain almost unchanged compared to the as-grown samples, with no observable peak shift or broadening, indicating no significant oxidation during the stacking process.[21, 22]

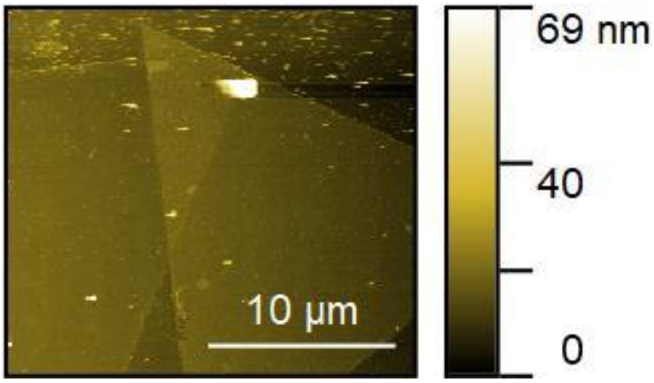

Supporting Information Figure. S4.| AFM images taken on monolayer the Van-der-Waals WS_2/MoS_2 stack edges.

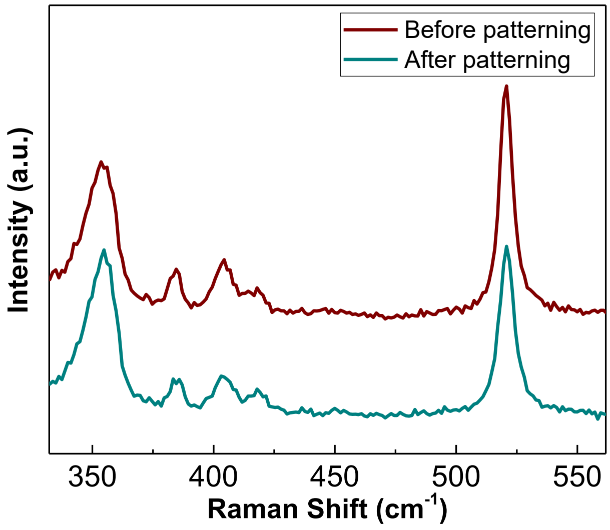

Supporting Information Figure. S5.| Raman spectroscopy of lithographically patterned ribbons showing no peak shift when comparing before and after edge exposure. This suggests that the patterning process does not induce significant strain or charge transfer into the 2D material

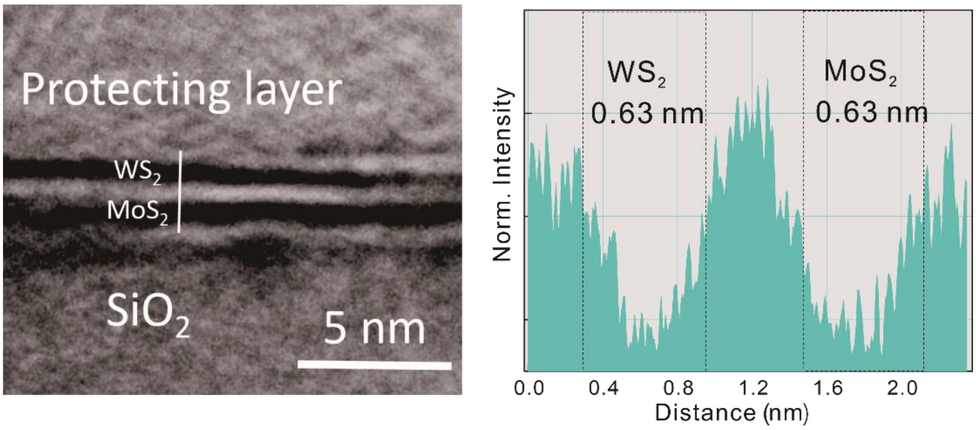

Supporting Information Figure. S6.| Cross-sectional HR-TEM image of WS_2/MoS_2 layers. Heterostack edges samples for cross-sectional TEM were crafted using focused ion beam (FIB), revealing the well-ordered stacking of MoS_2 and WS_2 homogeneous vdW stack edges with smooth and clear heterointerfaces.The linear profile indicated by a white line confirms the formation of a TMD heterostructure, with an interlayer spacing of 0.63nm, consistent with prior research.[23]

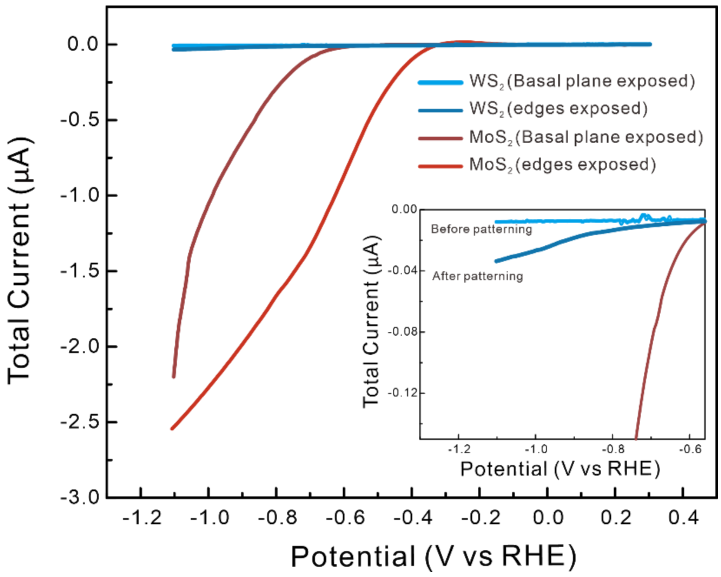

Supporting Information Figure. S7.| HER reaction current comparison. The electrochemical activity of 2D materials is significantly boosted after edge exposure, with WS_2 stack edges showing an approximately threefold increase in total HER current compared to their basal planes, while an-der-Waals MoS_2 edges exhibit a remarkable 260-fold increase in total current compared to the MoS_2 basal plane.

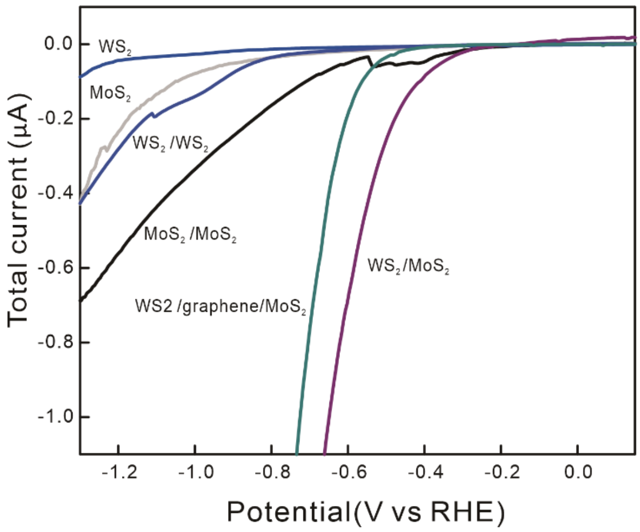

Supporting Information Figure. S8.| HER Polarization curves of the resulting samples (WS_2/MoS_2, WS_2/graphene/MoS_2, MoS_2/MoS_2, WS_2/WS_2, MoS_2, WS_2).

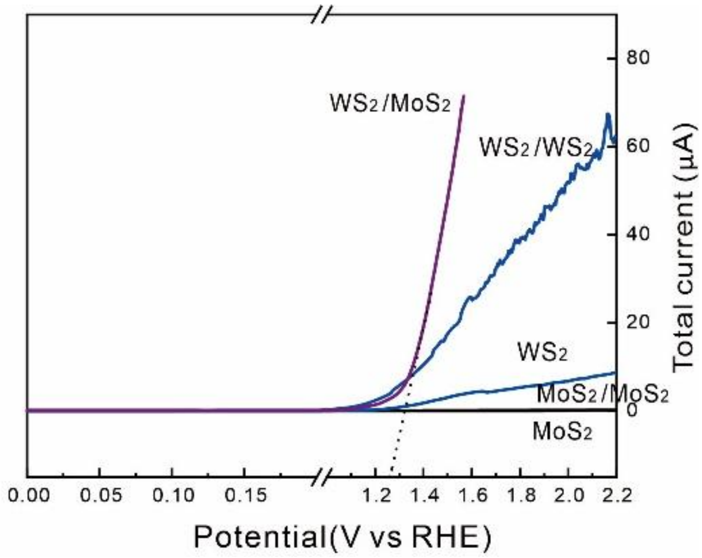

Supporting Information Figure. S9.| OER Polarization curves of the resulting samples (WS_2/MoS_2, WS_2/WS_2, WS_2, MoS_2/MoS_2, MoS_2). The onset potential for the WS_2/MoS_2 edges was extracted as 1.27 V.

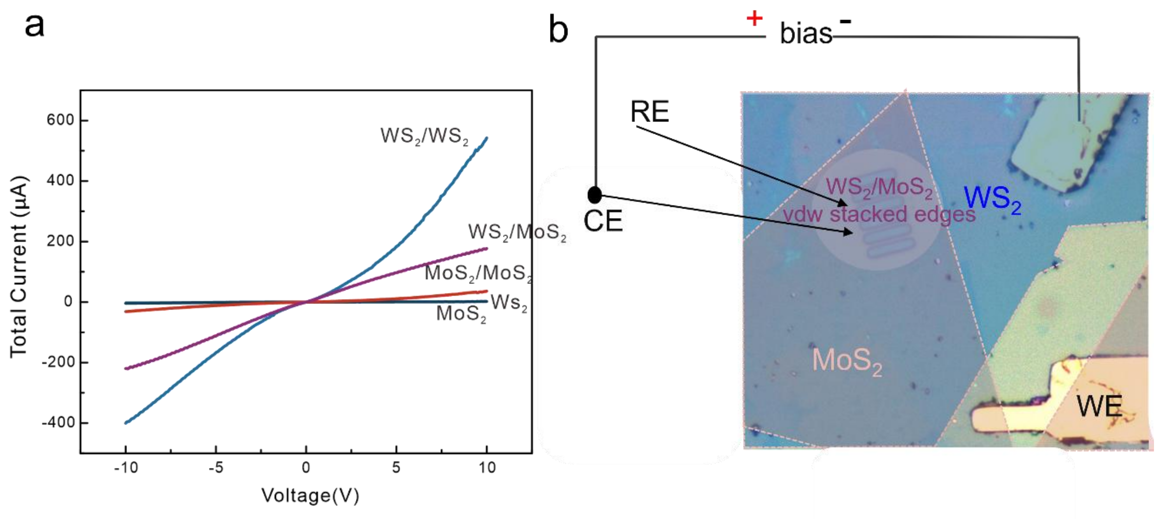

Supporting Information Figure. S10.| Fabrication and electrical properties of electrically contacted vdw edges (a)Current-voltage characteristics of different vdW stacks edges. (b) Set up of electrically contacted vdW edges to conduct HER at different potential differences: We used a standard microdroplet-based three-electrode setup to obtain HER polarization curves relative to the MoS_2 potential at different potential differences. Photolithography and lift-off procedures were employed to fabricate additional gold electrodes on the TMD material for applying the bias to the vdW-stacked edges. The bias output was controlled using a Keysight B2912A semiconductor analyzer, with one end connected to the material and the other to the counter electrode (CE).

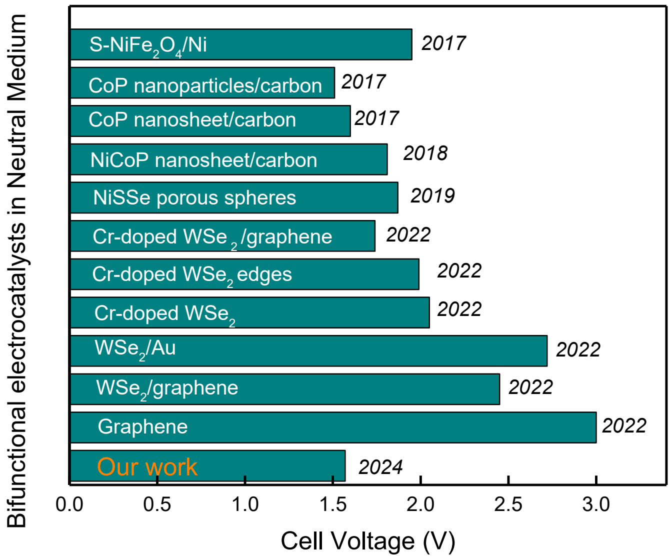

Supporting Information Figure. S11.| Cell Voltage for overall water splitting in Neutral medium: Comparison with previous reports on bifunctional catalysts[24-29].

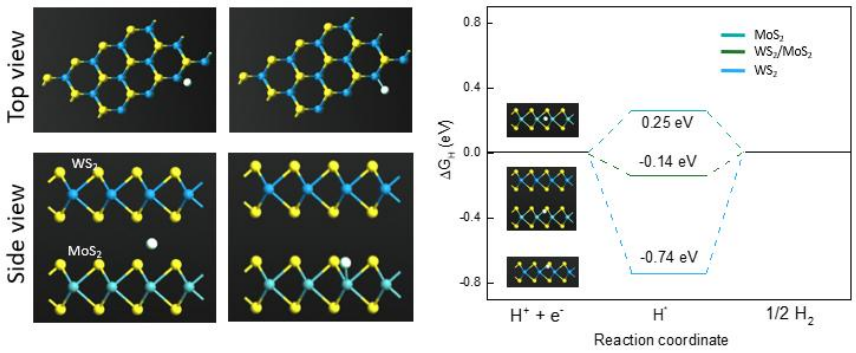

Supporting Information Figure. S12. To gain insight into the reaction pathways, we calculated the hydrogen adsorption free energies, we calculated the adsorption free energies of hydrogen atoms (ΔG_H*), comparing MoS_2, WS_2, and WS_2/MoS_2 van der Waals-stacked edges. The ΔG_H* values for MoS_2 was determined to be 0.25 eV, consistent with previous studies.[30] Notably, the WS_2/MoS_2 heterostack edge exhibited a ΔG_H* of -0.14 eV , significantly better than either MoS_2 or WS_2 alone, indicating enhanced hydrogen adsorption and desorption kinetics during the Volmer and Tafel steps.

Strategy	Tafel slope (m $Vdec^{-1}$)	reference
WS2/MoS_2 vdw stacked edges	*91.4*	*Our work*
WS2/MoS_2 vdw stacked edges (electric field-assisted)	*52.1*	
pristine	200	[31]
pristine	110	[32]
pristine	95	[32]
pristine	191	[33]
pristine	165	[34]
pristine	98	[35]
pristine	187	[36]
pristine	325	[37]
pristine	118	[38]
pristine	229	[39]
pristine	237	[40]
pristine	98	[41]
pristine	169	[42]
pristine	152	[43]
pristine	151	[44]
pristine	200	[45]
pristine	115	[46]
pristine	160	[47]
edge rich	136	[31]
edge rich	95	[32]
edge rich	96	[33]
edge rich	122	[36]
edge rich	109	[36]
edge rich	89	[38]
edge rich	163	[39]
edge rich	93	[40]
edge rich	79	[42]
atomic vacancies	105	[34]
atomic vacancies	90	[35]
atomic vacancies	67	[41]
atomic vacancies	86	[48]
atomic vacancies	102	[44]

annealing	89	[34]
annealing	117	[37]
plasma-functionalized	108	[49]
plasma-functionalized	105	[49]
plasma-functionalized	171	[37]
electric-field assisted	110	[45]
electric-field assisted	100	[46]

Supporting Information Table 1. Comparison of HER Tafel slope for 2H-MoS_2 after various modification strategies.

References

(1) Chen, D.-R.; Muthu, J.; Guo, X.-Y.; Chin, H.-T.; Lin, Y.-C.; Haider, G.; Ting, C.-C.; Kalbáč, M.; Hofmann, M.; Hsieh, Y.-P. Edge-dominated hydrogen evolution reactions in ultra-narrow MoS 2 nanoribbon arrays. *Journal of Materials Chemistry A* **2023**, *11* (29), 15802-15810.
(2) Chen, Y.-S.; Chiu, S.-K.; Tsai, D.-L.; Liu, C.-Y.; Ting, H.-A.; Yao, Y.-C.; Son, H.; Haider, G.; Kalbáč, M.; Ting, C.-C. Mediator-assisted synthesis of WS2 with ultrahigh-optoelectronic performance at multi-wafer scale. *npj 2D Materials and Applications* **2022**, *6* (1), 54.
(3) Yao, Y.-C.; Wu, B.-Y.; Chin, H.-T.; Yen, Z.-L.; Ting, C.-C.; Hofmann, M.; Hsieh, Y.-P. Nitrogen Pretreatment of Growth Substrates for Vacancy-Saturated MoS2. *ACS Applied Materials & Interfaces* **2023**, *15* (36), 42746-42752.
(4) Shindo, D.; Oikawa, T.; Shindo, D.; Oikawa, T. Energy dispersive x-ray spectroscopy. *Analytical electron microscopy for materials science* **2002**, 81-102.
(5) Muthu, J.; Khurshid, F.; Chin, H.-T.; Yao, Y.-C.; Hsieh, Y.-P.; Hofmann, M. The HER performance of 2D materials is underestimated without morphology correction. *Chemical Engineering Journal* **2023**, *465*, 142852.
(6) Huang, Y.; Nielsen, R. J.; Goddard III, W. A.; Soriaga, M. P. The reaction mechanism with free energy barriers for electrochemical dihydrogen evolution on MoS2. *Journal of the American Chemical Society* **2015**, *137* (20), 6692-6698.
(7) Abidi, N.; Bonduelle-Skrzypczak, A.; Steinmann, S. N. How stable are 2H-MoS2 edges under hydrogen evolution reaction conditions? *The Journal of Physical Chemistry C* **2021**, *125* (31), 17058-17067.
(8) Zeng, H.; Liu, G.-B.; Dai, J.; Yan, Y.; Zhu, B.; He, R.; Xie, L.; Xu, S.; Chen, X.; Yao, W. Optical signature of symmetry variations and spin-valley coupling in atomically thin tungsten dichalcogenides. *Scientific reports* **2013**, *3* (1), 1608.

(9) Yan, R.; Simpson, J. R.; Bertolazzi, S.; Brivio, J.; Watson, M.; Wu, X.; Kis, A.; Luo, T.; Hight Walker, A. R.; Xing, H. G. Thermal conductivity of monolayer molybdenum disulfide obtained from temperature-dependent Raman spectroscopy. *ACS nano* **2014**, *8* (1), 986-993.
(10) Luo, R.; Xu, W. W.; Zhang, Y.; Wang, Z.; Wang, X.; Gao, Y.; Liu, P.; Chen, M. Van der Waals interfacial reconstruction in monolayer transition-metal dichalcogenides and gold heterojunctions. *Nature Communications* **2020**, *11* (1), 1011.
(11) Yamamoto, M.; Einstein, T. L.; Fuhrer, M. S.; Cullen, W. G. Anisotropic etching of atomically thin MoS2. *The Journal of Physical Chemistry C* **2013**, *117* (48), 25643-25649.
(12) Xu, S.; Gao, X.; Hu, M.; Sun, J.; Jiang, D.; Zhou, F.; Liu, W.; Weng, L. Nanostructured WS2–Ni composite films for improved oxidation, resistance and tribological performance. *Applied surface science* **2014**, *288*, 15-25.
(13) Asgary, S.; Ramezani, A. H.; Ebrahimi Nejad, Z. Characterization of high quality, monolayer WS2 domains via chemical vapor deposition technique. *Applied Physics A* **2022**, *128* (2), 139.
(14) Hong, X.; Kim, J.; Shi, S.-F.; Zhang, Y.; Jin, C.; Sun, Y.; Tongay, S.; Wu, J.; Zhang, Y.; Wang, F. Ultrafast charge transfer in atomically thin MoS2/WS2 heterostructures. *Nature nanotechnology* **2014**, *9* (9), 682-686.
(15) Zhou, J.; Qin, J.; Guo, L.; Zhao, N.; Shi, C.; Liu, E.-z.; He, F.; Ma, L.; Li, J.; He, C. Scalable synthesis of high-quality transition metal dichalcogenide nanosheets and their application as sodium-ion battery anodes. *Journal of Materials Chemistry A* **2016**, *4* (44), 17370-17380.
(16) Chen, D.-R.; Muthu, J.; Guo, X.-Y.; Chin, H.-T.; Lin, Y.-C.; Haider, G.; Ting, C.-C.; Kalbáč, M.; Hofmann, M.; Hsieh, Y.-P. Edge-dominated hydrogen evolution reactions in ultra-narrow MoS 2 nanoribbon arrays. *Journal of Materials Chemistry A* **2023**.
(17) Park, S.; Garcia-Esparza, A. T.; Abroshan, H.; Abraham, B.; Vinson, J.; Gallo, A.; Nordlund, D.; Park, J.; Kim, T. R.; Vallez, L. Operando study of thermal oxidation of monolayer MoS2. *Advanced Science* **2021**, *8* (9), 2002768.
(18) Ghasemi, F.; Mohajerzadeh, S. Sequential solvent exchange method for controlled exfoliation of MoS2 suitable for phototransistor fabrication. *ACS applied materials & interfaces* **2016**, *8* (45), 31179-31191.
(19) Seo, D. B.; Kim, J.; Jo, Y. M.; Kim, D. I.; Lim, T. G.; Kang, S.; Yim, S.; Lee, S. S.; Kim, E. T.; An, K. S. MoS2–WS2 Heterostructures with Vertical Nanosheets for Enhanced Photocatalytic Hydrogen Generation through Morphology-Controlled Chemical Vapor Deposition. *Energy & Environmental Materials*, e70055.

(20) Dutta, A.; Krishnappa, M.; Porat, H.; Lavi, R.; Lal, A.; Yadav, M. K.; Mandić, V.; Makrinich, G.; Laikhtman, A.; Zak, A. Plasma-treated 1D transition metal dichalcogenides for efficient electrocatalytic hydrogen evolution reaction. *Journal of Materials Chemistry A* **2024**, *12* (37), 25176-25185.
(21) Woo, G.; Kim, H.-U.; Yoo, H.; Kim, T. Recyclable free-polymer transfer of nano-grain MoS2 film onto arbitrary substrates. *Nanotechnology* **2020**, *32* (4), 045702.
(22) Lin, G.; Zhao, M.-Q.; Jia, M.; Zhang, J.; Cui, P.; Wei, L.; Zhao, H.; Johnson, A. C.; Gundlach, L.; Zeng, Y. Performance enhancement of monolayer MoS2 transistors by atomic layer deposition of high-k dielectric assisted by Al2O3 seed layer. *Journal of Physics D: Applied Physics* **2019**, *53* (10), 105103.
(23) Naqi, M.; Kang, M. S.; Liu, N.; Kim, T.; Baek, S.; Bala, A.; Moon, C.; Park, J.; Kim, S. Multilevel artificial electronic synaptic device of direct grown robust MoS2 based memristor array for in-memory deep neural network. *npj 2D Materials and Applications* **2022**, *6* (1), 53.
(24) Chiang, C.-H.; Yang, Y.-C.; Lin, J.-W.; Lin, Y.-C.; Chen, P.-T.; Dong, C.-L.; Lin, H.-M.; Chan, K. M.; Kao, Y.-T.; Suenaga, K. Bifunctional monolayer WSe2/graphene self-stitching heterojunction microreactors for efficient overall water splitting in neutral medium. *ACS nano* **2022**, *16* (11), 18274-18283.
(25) Wu, R.; Xiao, B.; Gao, Q.; Zheng, Y. R.; Zheng, X. S.; Zhu, J. F.; Gao, M. R.; Yu, S. H. A janus nickel cobalt phosphide catalyst for high-efficiency neutral-pH water splitting. *Angewandte Chemie* **2018**, *130* (47), 15671-15675.
(26) Liu, T.; Xie, L.; Yang, J.; Kong, R.; Du, G.; Asiri, A. M.; Sun, X.; Chen, L. Self-standing CoP nanosheets array: a three-dimensional bifunctional catalyst electrode for overall water splitting in both neutral and alkaline media. *ChemElectroChem* **2017**, *4* (8), 1840-1845.
(27) Xue, Z. H.; Su, H.; Yu, Q. Y.; Zhang, B.; Wang, H. H.; Li, X. H.; Chen, J. S. Janus Co/CoP nanoparticles as efficient Mott–Schottky electrocatalysts for overall water splitting in wide pH range. *Advanced Energy Materials* **2017**, *7* (12), 1602355.
(28) Zeng, L.; Sun, K.; Chen, Y.; Liu, Z.; Chen, Y.; Pan, Y.; Zhao, R.; Liu, Y.; Liu, C. Neutral-pH overall water splitting catalyzed efficiently by a hollow and porous structured ternary nickel sulfoselenide electrocatalyst. *Journal of materials chemistry A* **2019**, *7* (28), 16793-16802.
(29) Liu, J.; Zhu, D.; Ling, T.; Vasileff, A.; Qiao, S.-Z. S-NiFe2O4 ultra-small nanoparticle built nanosheets for efficient water splitting in alkaline and neutral pH. *Nano Energy* **2017**, *40*, 264-273.

(30) He, Y.; Tang, P.; Hu, Z.; He, Q.; Zhu, C.; Wang, L.; Zeng, Q.; Golani, P.; Gao, G.; Fu, W. Engineering grain boundaries at the 2D limit for the hydrogen evolution reaction. *Nature communications* **2020**, *11* (1), 57.
(31) Zhang, R.; Zhang, M.; Yang, H.; Li, G.; Xing, S.; Li, M.; Xu, Y.; Zhang, Q.; Hu, S.; Liao, H. Creating Fluorine-Doped MoS2 Edge Electrodes with Enhanced Hydrogen Evolution Activity. *Small Methods* **2021**, *5* (11), 2100612.
(32) Zhu, J.; Wang, Z. C.; Dai, H.; Wang, Q.; Yang, R.; Yu, H.; Liao, M.; Zhang, J.; Chen, W.; Wei, Z. Boundary activated hydrogen evolution reaction on monolayer MoS2. *Nat. Commun.* **2019**, *10* (1), 1-7.
(33) Wang, Z.; Li, Q.; Xu, H.; Dahl-Petersen, C.; Yang, Q.; Cheng, D.; Cao, D.; Besenbacher, F.; Lauritsen, J. V.; Helveg, S. Controllable etching of MoS2 basal planes for enhanced hydrogen evolution through the formation of active edge sites. *Nano Energy* **2018**, *49*, 634-643.
(34) Xu, J.; Shao, G.; Tang, X.; Lv, F.; Xiang, H.; Jing, C.; Liu, S.; Dai, S.; Li, Y.; Luo, J. Frenkel-defected monolayer MoS2 catalysts for efficient hydrogen evolution. *Nat. Commun.* **2022**, *13* (1), 1-8.
(35) Li, H.; Tsai, C.; Koh, A. L.; Cai, L.; Contryman, A. W.; Fragapane, A. H.; Zhao, J.; Han, H. S.; Manoharan, H. C.; Abild-Pedersen, F. Activating and optimizing MoS2 basal planes for hydrogen evolution through the formation of strained sulphur vacancies. *Nat. Mater.* **2016**, *15* (1), 48-53.
(36) Su, S.; Zhou, Q.; Zeng, Z.; Hu, D.; Wang, X.; Jin, M.; Gao, X.; Nötzel, R.; Zhou, G.; Zhang, Z. Ultrathin alumina mask-assisted nanopore patterning on monolayer MoS2 for highly catalytic efficiency in hydrogen evolution reaction. *ACS Appl. Mater. Interfaces.* **2018**, *10* (9), 8026-8035.
(37) Ye, G.; Gong, Y.; Lin, J.; Li, B.; He, Y.; Pantelides, S. T.; Zhou, W.; Vajtai, R.; Ajayan, P. M. Defects engineered monolayer MoS2 for improved hydrogen evolution reaction. *Nano Lett.* **2016**, *16* (2), 1097-1103.
(38) Nguyen, A. D.; Nguyen, T. K.; Le, C. T.; Kim, S.; Ullah, F.; Lee, Y.; Lee, S.; Kim, K.; Lee, D.; Park, S. Nitrogen-plasma-treated continuous monolayer MoS2 for improving hydrogen evolution reaction. *ACS omega* **2019**, *4* (25), 21509-21515.
(39) Zhou, Q.; Luo, X.; Li, Y.; Nan, Y.; Deng, H.; Ou, E.; Xu, W. A feasible and environmentally friendly method to simultaneously synthesize MoS2 quantum dots and pore-rich monolayer MoS2 for hydrogen evolution reaction. *Int. J. Hydrog. Energy* **2020**, *45* (1), 433-442.
(40) Zhou, Q.; Su, S.; Cheng, P.; Hu, X.; Gao, X.; Zhang, Z.; Liu, J. M. Vertically conductive MoS 2 pyramids with a high density of active edge sites for efficient hydrogen evolution. *J. Mater. Chem. C* **2020**, *8* (9), 3017-3022.

(41) Pető, J.; Ollár, T.; Vancsó, P.; Popov, Z. I.; Magda, G. Z.; Dobrik, G.; Hwang, C.; Sorokin, P. B.; Tapasztó, L. Spontaneous doping of the basal plane of MoS2 single layers through oxygen substitution under ambient conditions. *Nat. Chem.* **2018**, *10* (12), 1246-1251.
(42) Bala, A.; Sen, A.; Kim, Y. H.; Kim, Y. M.; Gandla, S.; Park, H.; Kim, S. Large-Area MoS2 Nanosheets with Triangular Nanopore Arrays as Active and Robust Electrocatalysts for Hydrogen Evolution. *J. Phys. Chem. C.* **2022**.
(43) Cho, H. Y.; Nguyen, T. K.; Ullah, F.; Yun, J. W.; Nguyen, C. K.; Kim, Y. S. Salt-assisted clean transfer of continuous monolayer MoS2 film for hydrogen evolution reaction. *Phys. B: Condens. Matter.* **2018**, *532*, 84-89.
(44) Tsai, C.; Li, H.; Park, S.; Park, J.; Han, H. S.; Nørskov, J. K.; Zheng, X.; Abild-Pedersen, F. Electrochemical generation of sulfur vacancies in the basal plane of MoS2 for hydrogen evolution. *Nat. Commun.* **2017**, *8* (1), 1-8.
(45) Wang, J.; Yan, M.; Zhao, K.; Liao, X.; Wang, P.; Pan, X.; Yang, W.; Mai, L. Field effect enhanced hydrogen evolution reaction of MoS2 nanosheets. *Adv. Mater.* **2017**, *29* (7), 1604464.
(46) Wang, Y.; Udyavara, S.; Neurock, M.; Frisbie, C. D. Field effect modulation of electrocatalytic hydrogen evolution at back-gated two-dimensional MoS2 electrodes. *Nano Lett.* **2019**, *19* (9), 6118-6123.
(47) Tao, L.; Duan, X.; Wang, C.; Duan, X.; Wang, S. Plasma-engineered MoS2 thin-film as an efficient electrocatalyst for hydrogen evolution reaction. *ChemComm* **2015**, *51* (35), 7470-7473.
(48) Cho, H.-Y.; Nguyen, T. K.; Ullah, F.; Yun, J. W.; Nguyen, C. K.; Kim, Y. S. Salt-assisted clean transfer of continuous monolayer MoS2 film for hydrogen evolution reaction. *Physica B: Condensed Matter* **2018**, *532*, 84-89.
(49) Tao, L.; Duan, X.; Wang, C.; Duan, X.; Wang, S. Plasma-engineered MoS 2 thin-film as an efficient electrocatalyst for hydrogen evolution reaction. *ChemComm* **2015**, *51* (35), 7470-7473.
